# Type 2 Diabetes Is Associated with Reduced ATP-Binding Cassette Transporter A1 Gene Expression, Protein and Function

**DOI:** 10.1371/journal.pone.0022142

**Published:** 2011-07-27

**Authors:** Dipesh C. Patel, Christiane Albrecht, Darrell Pavitt, Vijay Paul, Celine Pourreyron, Simon P. Newman, Ian F. Godsland, Jonathan Valabhji, Desmond G. Johnston

**Affiliations:** 1 Division of Medicine, Imperial College London, London, United Kingdom; 2 Oncology Drug Discovery and Women's Health Group, Faculty of Medicine, Imperial College London, London, United Kingdom; 3 Department of Physiology, Technical University of Munich, Munich, Germany; 4 Institute of Biochemistry and Molecular Medicine, University of Bern, Bern, Switzerland; 5 Centre of Oncology and Molecular Sciences, University of Dundee, Dundee, United Kingdom; University of South Florida, United States of America

## Abstract

**Objective:**

Increasing plasma glucose levels are associated with increasing risk of vascular disease. We tested the hypothesis that there is a glycaemia-mediated impairment of reverse cholesterol transport (RCT). We studied the influence of plasma glucose on expression and function of a key mediator in RCT, the ATP binding cassette transporter-A1 (ABCA1) and expression of its regulators, liver X receptor-α (LXRα) and peroxisome proliferator-activated receptor–γ (PPARγ).

**Methods and Results:**

Leukocyte *ABCA1*, *LXR*α and *PPARγ* expression was measured by polymerase chain reaction in 63 men with varying degrees of glucose homeostasis. ABCA1 protein concentrations were measured in leukocytes. In a sub-group of 25 men, ABCA1 function was quantified as apolipoprotein-A1-mediated cholesterol efflux from 2–3 week cultured skin fibroblasts. Leukocyte *ABCA1* expression correlated negatively with circulating HbA1c and glucose (rho = −0.41, p<0.001; rho = −0.34, p = 0.006 respectively) and was reduced in Type 2 diabetes (T2DM) (p = 0.03). Leukocyte ABCA1 protein was lower in T2DM (p = 0.03) and positively associated with plasma HDL cholesterol (HDL-C) (rho = 0.34, p = 0.02). Apolipoprotein-A1-mediated cholesterol efflux correlated negatively with fasting glucose (rho = −0.50, p = 0.01) and positively with HDL-C (rho = 0.41, p = 0.02). It was reduced in T2DM compared with controls (p = 0.04). These relationships were independent of *LXR*α and *PPARγ* expression.

**Conclusions:**

*ABCA1* expression and protein concentrations in leukocytes, as well as function in cultured skin fibroblasts, are reduced in T2DM. ABCA1 protein concentration and function are associated with HDL-C levels. These findings indicate a glycaemia- related, persistent disruption of a key component of RCT.

## Introduction

People with hyperglycaemia are at increased risk of coronary heart disease (CHD). The risk is high in type 2 diabetes (T2DM) (2–4 fold elevation) [1] and applies even to those with lesser degrees of glucose elevation (impaired glucose regulation). Even in the general population, there is a positive relationship between glucose (or HbA1c) levels and CHD rates.[2] Several underlying mechanisms have been proposed, including protein glycation and free radical damage.[3] Both diabetes and impaired glucose regulation are accompanied by dyslipidaemia, a major feature of which is low levels of circulating high density lipoprotein cholesterol (HDL-C). Impaired reverse cholesterol transport (RCT) has therefore been implicated.[4]

RCT is the process by which excess cholesterol is removed from the body. It begins in peripheral tissues when lipid-poor apolipoprotein A-I (apo-A1) induces cholesterol and phospholipid mobilisation from intracellular storage sites to the plasma membrane.[5] The membrane-associated ATP binding cassette transporter-A1 (ABCA1) is integral to the subsequent transmembrane lipid transfer to form nascent HDL.[6] Later cholesterol efflux to HDL is mediated by the related transporter, ATP binding cassette transporter-G1 (ABCG1) with some evidence of synergism.[7] Defects in *ABCA1* impair apo-A1-mediated lipid efflux from cells and *ABCA1* knockout mice develop early atherosclerosis.[8], [9] Functional *ABCA1* mutations in both Tangier disease and familial HDL deficiency lead to very low levels of circulating HDL, almost all of which is lipid-poor, as newly synthesised apo-A1 fails to acquire cholesterol and phospholipids.[10]

*ABCA1* expression in several tissues is up-regulated by oxysterol interaction with the liver X receptor-α (*LXR*α). Stimulation of *LXR*α transcription by peroxisome proliferator-activated receptor-γ (PPARγ) upregulates *ABCA1* expression and increases apo-A1-mediated cholesterol efflux from macrophages.[11]

*ABCA1* expression is decreased in the liver and peritoneal macrophages of diabetic compared to control mice and protein levels have been reported to be reduced in mouse models of diabetes.[12], [13] In human studies, fibroblast ABCAI function has been shown to be impaired by advanced glycation end products [14] and we have previously observed an inverse relationship between *ABCA1* expression in peripheral leucocytes and fasting glucose in healthy young and middle-aged men.[15] These findings suggested a potential mechanism for the hyperglycaemia-induced increased risk of early vascular disease. In the present study, we explore relationships between glycaemia, expression of *ABCA1* and *ABCG1,* ABCA1 protein concentrations and ABCA1 function in people with varying degrees of hyperglycaemia, and whether these relationships are influenced by *LXRα* or *PPARγ* expression.

## Methods

### Participants

Sixty three men aged over 18 years were recruited from regional metabolic clinics and primary care, with the assistance of the UK Diabetes Research Network. Participants included 18 with T2DM and 18 with impaired glucose regulation by World Health Organization criteria. The patients with T2DM were treated with diet alone and had not taken hypoglycaemic or lipid-lowering medication. Subjects taking systemic corticosteroids or medications with marked effects on insulin secretion were excluded. Patients were free of microvascular and macrovascular complications. 27 healthy, aged matched normoglycaemic controls were also studied following local advertisement.

### Ethics statement

Written consent was obtained and ethical approval for the study was from the Hammersmith, Queen Charlotte's and Chelsea Research Ethics Committee *(REC Reference 05/Q0406/33).* The study protocol conformed to the ethical guidelines of the 1975 Declaration of Helsinki.

### Procedures

After an overnight fast, a clinical and routine biochemical assessment was conducted. The control subjects and those without known diabetes underwent a 75g Oral Glucose Tolerance Test (OGTT) and were classified using the American Diabetes Association criteria (2003).[16] Fasting blood samples only were taken from patients with known T2DM. 25 participants, selected randomly, consented to undergo more detailed investigation which involved a 4 mm punch skin biopsy sample from the forearm under sterile conditions to enable functional analyses in skin fibroblasts.

### Laboratory measurements

Fasting glucose, creatinine and liver enzymes were measured using standard laboratory techniques. HbA1c (DCCT aligned) was measured by cation-exchange high performance liquid chromatography using a Tosoh Automated glycohaemoglobin analyser G7 (Tosoh Bioscience Inc., Tokyo, Japan). Cholesterol, lipoprotein and triglyceride measurements were performed using an Olympus AU 2700 autoanalyser (Olympus Life & Material Science, Ireland). LDL-cholesterol was calculated using the Friedewald formula. Plasma non-esterifed fatty acid (NEFA) concentrations were measured by an *in vitro* colorimetric method (Wako Chemicals, Neuss, Germany) using a Cobas Mira analyzer (Hoffman-LaRoche and company, Basel, Switzerland).

### Leukocyte isolation and gene expression

Peripheral leukocytes were isolated from 12 ml blood in subjects and frozen at −80°C after erythrocyte lysis (Qiagen, Crawley, UK). Total RNA extraction was performed using Qiashredder and RNeasy (Qiagen) according to manufacturer's protocol. Reverse transcription and multiplex quantitative PCR were performed using a high capacity cDNA reverse transcription kit and Taqman universal mastermix reagents (Applied Biosciences, Warrington, UK). Primer and probe sets were exon overlapping (Applied Biosystems). A Rotorgene thermal cycler (Corbett Life Science, Cambridge, UK) was used to measure gene expression. cDNA (30 ng) was amplified in a total volume of 10 µL. *β-Actin* was used as the housekeeper gene. All experiments were performed in duplicate. This technique was validated in our earlier study.[15] *ABCA1* expression results were further validated in 25 subjects using an additional housekeeper, glyceraldehyde 3-phosphate dehydrogenase (*GAPDH*). As results with GAPDH were very similar to those with *β-Actin*, only *β-*Actin derived data are described. Target gene quantity was calculated from a standard curve. Gene expression data from multiple experiments were compared to a calibrator sample and normalised to the house keeper gene.

### Leukocyte protein expression

Total cell lysates were prepared by incubating the leukocyte cell pellets (n = 50) with 1 ml ice-cold lysis buffer (50 mM mannitol, 2 mM EDTA, 50 mM, pH 7.6) containing complete protease inhibitor (Roche, Germany) and 0.1% w/v Triton X-100 for 60 minutes on ice. This was followed by five homogenization cycles (20 s each and 10 minutes resting time on ice after each homogenization step) in a Fastprep homogenizer. The leukocyte homogenates were centrifuged at 15,000 g for 15 minutes at 4°C and the supernatants containing clear cell lysates were collected. Protein concentrations were determined by a bicinchoninic acid assay.[17] The clear leukocyte cell lysates were stored at −80°C until ABCA1 protein measurement was performed. Frozen leukocyte lysates were thawed and ABCA1 protein concentration was measured using an ELISA method as previously described.[18] Briefly, either 50 µl ABCA1 peptide (ab14148, Abcam, UK) calibrators (range 8–1000 ng) or 50 µl of unknown leukocyte lysates were incubated in a ABCA1 peptide pre-coated and BSA blocked micro titre plate along with 100 µl (2 µg/l diluted in PBST) anti-ABCA1 rabbit antibody (ab7360, Abcam, UK). The incubation was carried out for 5 hours at room temperature while shaking on an ELISA plate shaker. The plate was washed with PBST and further processed by using biotin labelled anti-rabbit IgG goat antibody, streptavidin-HRP conjugate and tetramethylbenzidine enzyme substrate system. The enzymatic reaction was stopped by addition of sulphuric acid and absorbance measured at 450 nm on an ELISA plate reader (Sunrise, Tecan, Austria). ABCA1 protein concentrations of unknown samples were calculated from a calibration curve and results were expressed as ABCA1 protein ng/µg total protein.

### Fibroblast cell culture and cholesterol efflux measurement

Primary fibroblast cell lines were obtained from human dermal biopsy following enzymatic digestion in the sub-group of 25 subjects (9 controls, 6 with impaired regulation and 10 T2DM) who underwent a skin biopsy. Primary cells were cultured with DMEM (Invitrogen Ltd, Paisley, UK) which contained 25 mmol/l glucose, supplemented with 10% fetal bovine serum and 1% antimicrobial solution (Sigma-Aldrich Co, Poole, UK). The medium was changed twice weekly until cells were 80-100% confluent. After culture for 2–3 weeks, cells from passage 4 to 7 were used for cholesterol efflux measurements. Fibroblasts were plated in wells until 60–80% confluence. Cholesterol loading and efflux experiments were carried out following an accepted methodology using apo-A1.[19] In brief, cells were loaded with 0.5 µCi/ml 1,2-^3^[H]-cholesterol (Perkin Elmer, Boston, MA) for 24 hours in the presence of 30 µg/ml unlabelled cholesterol (Sigma-Aldrich Co.) to induce *ABCA1*. Cells were then washed with PBS/BSA 0.2% (v/v) and efflux was commenced using 10 µg/ml apo-A1 (Sigma-Aldrich Co.). After 20 hrs the medium was collected, centrifuged and radioactivity was measured using liquid scintillation counting (Beckman LS6000 Coulter UK Ltd, High Wycombe, UK). Cellular cholesterol was extracted using hexane/propanol (3∶2 v/v) and counted. Percentage cholesterol efflux was calculated by dividing the radioactive counts in the efflux medium by the sum of the counts in the media and cell extract. Experiments were performed in triplicate with a mean CV of 12%. Apo-A1-mediated lipid export was derived by subtraction of non-specific efflux (measured in control wells without apo-A1).

### Statistical analysis

Variation between the groups was compared by ANOVA, or Kruskal-Wallis test depending on variable distribution. Likewise, a t-test or Mann-Whitney test was used to compare individual groups Smoking status was analysed using a Chi squared test. Relationships between variables were assessed by Spearman correlation. Multiple linear regression was used to assess independence of relationships after log- transformation, where appropriate, to normalise distributions. A two sided p value <0.05 was considered significant. Sigmastat version 3.5 (Systat Software, Inc.) and Prism version 4 (Graphpad Software, Inc.) were used for analyses and graphical illustration, respectively.

## Results

As expected, patients with diabetes were heavier compared to controls and had a greater waist circumference (Table1). HDL-C was significantly lower in patients with diabetes, compared with controls, but other lipid parameters were not significantly different across subject groups. Standard laboratory renal, liver and thyroid function tests were normal in all subjects. HbA1c and fasting glucose have been used as the indices of glycaemia as they were measured in all subjects and they correlated strongly with each other (rho = 0.77, p<0.0001).

**Table 1:** Baseline participant demographic data.

Variable	Controls (n = 27)	IGH (n = 18)	DM (n = 18)	ANOVA (p)
Age, yrs	52±16	58±8	57±11	0.40
BMI, kg/m^2^	24.3±2.2	27.3±4.1	27.9±3.2*	<0.001
Waist circumference, cm	91 (85–96)	96 (87–105)	101 (94–105) *	0.003
Alcohol consumption,units	20 (4–24)	8 (2–18)	12 (2–20)	0.15
Smokers, %	3 (10)	6 (33)	4 (22)	0.192
Systolic BP, mmHg	132±13	138±17	131±14	0.30
Diastolic BP, mmHg	81±10	83±11	82±6	0.77
Fasting Glucose, mmol/l	5.10 (4.70–5.40)	5.56 (4.95–5.95)	6.8 (6.3–8.8) †	<0.001
HbA1c, %	5.5±0.4	5.9±0.5	7.3±1.5†	<0.001
2 hr Glucose, mmol/l	4.9±1.3	7.3±2.2*	-	<0.001
Total Cholesterol, mmol/l	5.38±0.89	5.14±1.06	5.03±1.10	0.49
HDL-C, mmol/l	1.53±0.29	1.32±0.38	1.23±0.26*	0.005
LDL-C, mmol/l	3.29±0.65	3.26±0.71	3.11±0.89	0.72
Triglycerides, mmol/l	1.17 (0.74–1.59)	0.97 (0.77–1.38)	1.37 (0.94–2.11)	0.18

Normally distributed variables are expressed as mean and standard deviation. Skewed data are represented by median (interquartile range).

Abbreviations: IGH = impaired glucose regulation; DM = type 2 diabetes; ANOVA or equivalent analysis of variance; *p<0.05 relative to controls, †p<0.05 relative to control and IGH subjects.

### ABCA1 gene expression and protein concentrations in peripheral blood leukocytes

**Figure 1:**
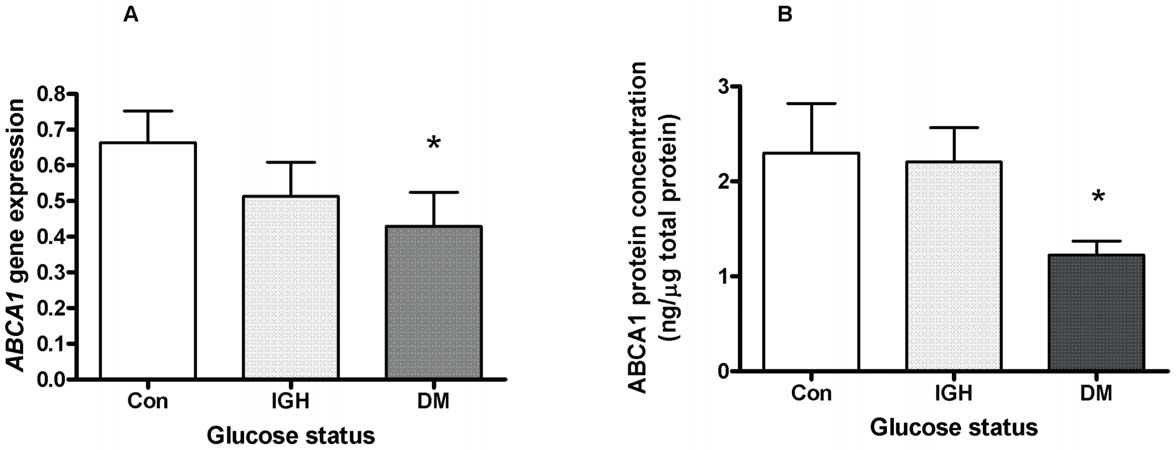
*ABCA1* gene expression and protein concentration in blood leukocytes is reduced in type 2 diabetes. A. Leukocyte *ABCA1* gene expression, normalised to housekeeper gene (mean and standard error) according to subject glycaemic status in 63 participants. Con = Controls (n = 27), IGH = Impaired glucose regulation (n = 18), DM = type 2 diabetes (n = 18). Con vs. DM; p = 0.03, Con vs. IGH; p = 0.27, IGH vs. DM p = 0.33 (* p<0.05). B. Leukocyte ABCA1 protein concentration (ng/µg protein) expressed as mean and standard error is illustrated according to subject glycaemic status in 50 participants. Con = Controls (n = 17), IGH  = Impaired glucose regulation (n = 17), DM = type 2 diabetes (n = 16) Con vs DM; p = 0.03, Con vs IGH; p = 1; IGH vs DM p = 0.06 (* p<0.05).

**Figure 2:**
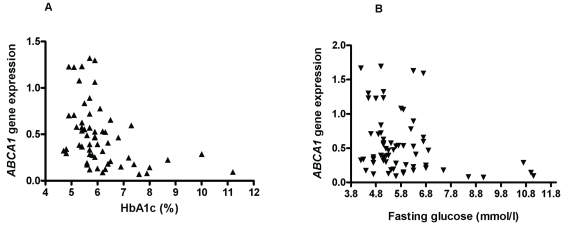
Leukocyte *ABCA1* gene expression is reduced with increasing glycaemia. A. Relationship of leukocyte *ABCA1* expression (normalised to housekeeper gene) to glycated haemoglobin (HbA1c) in 63 participants (27 control, 18 impaired glucose homeostasis and 18 type 2 diabetes) rho = −0.41, p<0.001. B. Relationship of leukocyte *ABCA1* expression (normalised to housekeeper gene) to fasting plasma glucose in 63 participants (27 control, 18 impaired glucose homeostasis and 18 type 2 diabetes) rho = −0.34, p = 0.006.

Leukocyte *ABCA1* expression was significantly lower in patients with T2DM compared with controls (p = 0.03; Figure 1A). In the combined groups, *ABCA1* expression fell with increasing HbA1c (rho = −0.41, p<0.001; Figure 2A) and with increasing fasting glucose levels (rho = −0.34, p = 0.006; Figure 2B).

There was no significant relationship between leukocyte *ABCA1* expression and age, body mass index or waist circumference. Leukocyte ABCA1 protein concentration was lower in patients with T2DM compared with controls (p = 0.03; Figure 1B) although the relationship with fasting glucose was not significant statistically (rho = −0.25, p = 0.08). No significant relationship was observed between plasma NEFA levels and *ABCA1* expression (p = 0.40).

### Relationship of ABCG1 expression with glycaemia and ABCA1 expression

Leukocyte *ABCA1* and *ABCG1* expression were positively related (rho = 0.40, p = 0.005, Figure S1). Leukocyte *ABCG1* expression in patients with T2DM was not significantly reduced (p = 0.08) and there were no differences between other groups. There was a borderline negative relationship between leukocyte *ABCG1* expression and HbA1c (rho = −0.29, p = 0.05) Figure S1, but not fasting glucose (p = 0.35). Analysis of *ABCA1* expression and glycaemia (mean glucose or HbA1c) using a linear model, demonstrated that only *ABCA1* expression remained a significant predictor of *ABCG1* expression.

### Apolipoprotein-A1 mediated cholesterol efflux in skin fibroblasts and glycaemia

**Figure 3:**
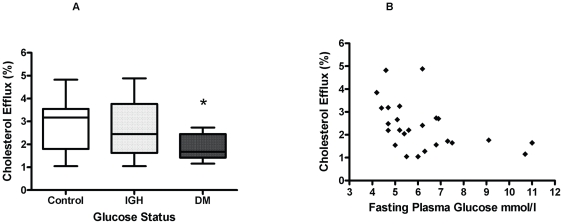
Cholesterol efflux in skin fibroblasts is reduced in type 2 diabetes and with increasing blood glucose concentration. A. Cholesterol efflux to Apo-A1 according to glycaemic status in a subgroup of 25 men (Mean and Standard error). 0.5 µCi/ml 1,2-^3^[H]-cholesterol was loaded onto cells for 24 hours in the presence of 30 µg/ml unlabelled cholesterol. After equilibration, apo-A1 (10 µg/ml) was added to initiate efflux and incubated for 20 hours. Control wells were incubated with culture media alone and apo-A1 mediated efflux was calculated by subtracting background efflux from efflux mediated by apo-A1. Con = Control (n = 9) IGH = Impaired glucose regulation (n = 6), DM = type 2 diabetes (n = 10). Con versus DM; p = 0.04, Con vs. IGH; p = 0.39 (* p<0.05). B. Relationship between fasting glucose and fibroblast apo-AI-mediated cholesterol efflux. rho = −0.50, p = 0.01.

Apo-A1-mediated cholesterol efflux from fibroblasts was reduced by 36% in diabetes (p = 0.04; Figure 3A). After adjusting for protein concentration, efflux remained significantly lower in patients with diabetes compared to healthy men (4.5% versus 11.2%, p = 0.008). Efflux fell with increasing fasting plasma glucose (rho = −0.50, p = 0.01; Figure 3B). The inverse relationship with HbA1c was not statistically significant (rho = −0.30, p = 0.07). There was no significant relationship between apo-A1-mediated cholesterol efflux and age nor with body weight. Apo-A1-mediated cholesterol efflux from fibroblasts fell with increasing waist circumference (r = −0.53, p<0.01) but bivariate analysis demonstrated this relationship to be dependent on fasting plasma glucose.

### Relationship of ABCA1 with LXRα and PPARγ

There was no significant relationship between fasting plasma glucose nor HbA1c levels and either leukocyte *PPARγ* (versus HbA1c p = 0.15, Figure S3) or *LXRα* (versus HbA1c p = 0.87, Figure S2) expression. Leukocyte *LXRα* expression was positively related to leukocyte *ABCA1* (r = 0.40, p = 0.002), Figure S2, as well as *ABCG1* (rho = 0.67, p<0.001) expression, but there were no such relationship between leukocyte *ABCA1* and *PPARγ* expression (Figure S3). Using bivariate linear regression, fasting plasma glucose concentrations and leukocyte *LXRα* expression both remained independent predictors of leukocyte *ABCA1* gene expression (p = 0.005 and p = 0.001 respectively). Neither *LXRα* expression nor glycaemia were significant independent contributors to *ABCG1* expression.

### Relationship of ABCA1 to plasma HDL cholesterol

**Figure 4:**
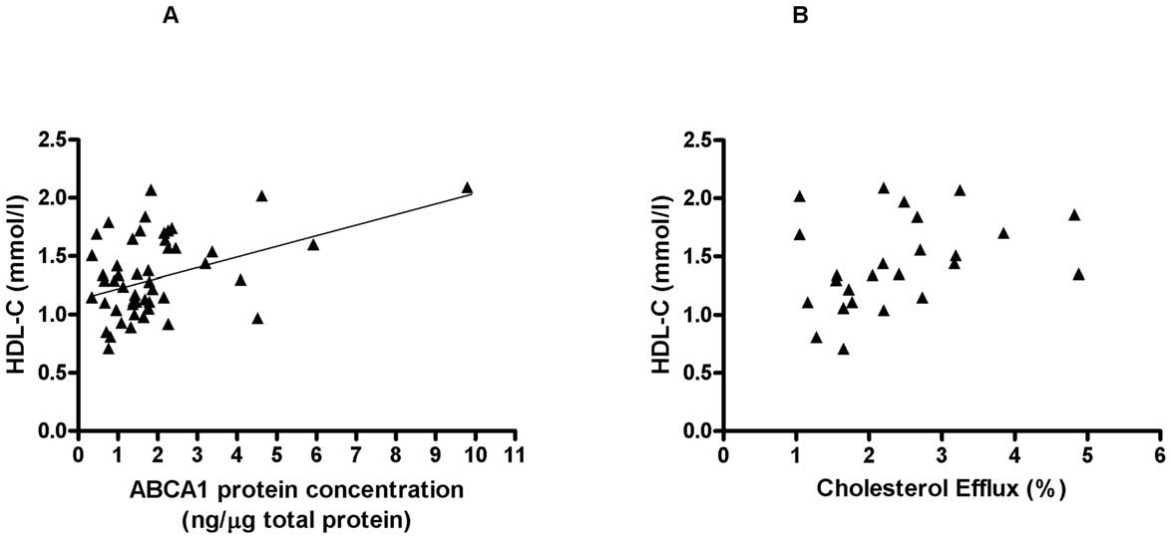
Positive relationship of human ABCA1 measurements with plasma HDL-C concentration. A. ABCA1 protein concentration in leukocytes positively associates with plasma HDL-C concentration. n = 50, rho = 0.34, p = 0.02. B. Cholesterol efflux to apo-A1 positively relates with plasma HDL-C concentrations. Relationship between ABCA1-mediated cholesterol efflux in fibroblasts (mean of triplicate experiments) and plasma HDL-C concentration. n = 25, rho = 0.41, p = 0.02.

Leukocyte *ABCA1* and *ABCG1* expression did not significantly associate with plasma HDL-C concentrations. Leukocyte ABCA1 protein concentrations correlated positively and independently with circulating plasma HDL-C levels (rho = 0.34, p = 0.02; Figure 4A). Cholesterol efflux related positively to plasma HDL-C concentrations (rho = 0.41, p = 0.02; Figure 4B).

## Discussion

We have demonstrated that *ABCA1* gene expression, protein concentrations and transporter function are reduced in drug naive men with T2DM. Gene expression and protein concentrations were reduced in blood leukocytes, and cellular cholesterol removal to Apo-A1 (which exclusively involves ABCA1) was reduced in skin fibroblasts. These relationships were independent of variation in *LXRα* or *PPARγ* expression. These observations suggest novel mechanisms whereby hyperglycaemia could adversely influence cellular cholesterol metabolism. Such mechanisms could contribute to the well-established association between elevated glucose levels and risk of vascular disease.

*ABCA1* is highly expressed in macrophages. Ideally, *ABCA1* expression and protein content should be studied in arterial wall macrophages, but this was not possible for ethical reasons. Leukocyte *ABCA1* RNA levels in humans reflect *ABCA1* expression in circulating monocytes and can be used as a marker for this.[20] It should be noted that gene expression increases 4-fold in monocytes during differentiation into macrophages.[21] Peripheral blood leukocytes have the advantage of being readily obtained from large numbers of subjects and leukocyte *ABCA1* has proven a useful surrogate in other clinical situations where the variations in *ABCA1* expression have been observed in man.[22]

Leukocyte ABCA1 has not been previously considered to influence plasma HDL or RCT directly.[20], [23] Recent data, however, have suggested that cells other than macrophages influence tissue cholesterol removal (eg adipocytes).[24] Leukocyte ABCA1 is known to protect against atherosclerosis in animals [25], but our observations cannot distinguish between possible direct and indirect effects of leukocyte ABCA1 on lipid transport.

*ABCA1* expression was decreased in leukocytes from patients with T2DM and was directly related to the level of glycaemia. Most of the decline in gene expression occurred up to an HbA1c level of 7.5%, with little further fall above this value. Our *ABCA1* expression data are compatible with and extend our previous findings in healthy men.[15] We further demonstrate concordant changes in leukocyte *ABCA1* expression and protein concentrations in patients with T2DM. Our leukocyte expression results appear to contrast with the findings reported by *Hoang* and colleagues who did not find a difference in leukocyte *ABCA1* expression between patients with diabetes and controls.[22] This previous study was conducted in a smaller cohort with one third of patients receiving hypoglycaemic medications. ABCA1 function was assessed on the basis of the ability of the subject's plasma to induce cholesterol efflux from human macrophages cell lines. [22] There were baseline differences in lipid parameters which may have had an effect on the capacity of plasma to induce cholesterol efflux. [22] Our findings are compatible with previous data in monocytes, where reduced *ABCA1* expression was observed in patients with diabetes and dyslipidaemia.[26]

Our results contrast with studies that showed reduced expression and function of ABCG1 (but not ABCA1) in monocytes and macrophages in people with T2DM [27], [28], but these earlier studies were not designed or conducted in drug naive patients who were free of complications. In the study by *Mauldin* and colleagues [28], information pertaining to age, other medical conditions and drug treatments was unavailable. Forty percent of participants studied by *Zhou* et al., had evidence of retinopathy or nephropathy and all were receiving either oral hypoglycaemic treatments or insulin treatment.[27] It is feasible that hypoglycaemic treatments may have contributed to the observed discrepancies in the relationship between circulating glucose and *ABCG1* expression. For example, insulin decreases human macrophage *ABCA1* and *ABCG1* gene expression *in vitro.* [29] Animal studies indicate that complications such as nephropathy independently reduce macrophage ABCA1 and increase cellular cholesterol content.[30] Treatment of nephropathy in this study restored ABCA1-mediated cholesterol efflux in macrophages.[30] The present study has the advantage of having matched controls and patients who were drug naive and free of diabetes related complications.

Skin fibroblasts were used to assess ABCA1 function in our study. Skin fibroblasts have been previously employed for this purpose, where functional consequences of *ABCA1* genetic variants have been studied.[8] Our tritiated labelled cholesterol-based technique assessed specific apo-A1-mediated cholesterol efflux from intracellular and membranous sites to the extracellular medium. This was derived from a validated methodology.[19]

It should be noted that our gene expression measurements in leukocytes and cholesterol efflux measurements in fibroblasts were made against very different backgrounds. Leukocyte gene expression is likely to relate to conditions under which the leukocytes were taken at the time of sampling, whereas fibroblasts had undergone prolonged *in vitro* culture. Despite the prolonged *in vitro* conditions at the high glucose levels (25mmol/l) present in the culture medium, a relationship was apparent between fibroblast cholesterol efflux and the *in vivo* glucose concentrations which pertained at the time of sampling. High medium glucose levels have not influenced *ABCA1* expression in other studies of leucocytes [31] and the results imply a prolonged metabolic memory on the part of the cells in humans. Longitudinal studies in both type I and type 2 diabetes have demonstrated that intensive glucose lowering leads to fewer cardiovascular disease events many years later, indicating a possible “legacy” effect.[32], [33] The mechanisms underlying this prolonged benefit are unknown. The preserved effect of glycaemia, at the time of sampling, on ABCA1 function in fibroblasts in our study may indicate a possible mechanism.

We have shown that ABCA1-mediated cholesterol efflux is positively related to plasma HDL-C concentration, compatible with its key role in HDL formation. This positive relationship between ABCA1 function and blood HDL-C concentrations has previously only been described in humans with known ABCA1 mutations.[34] and was not observed in fibroblasts taken from patients with the metabolic syndrome.[35] It had been thought that increased intravascular HDL remodelling and catabolism were the cause of lower HDL-C concentrations in patients with type 2 diabetes [36] but our observations suggest that reduced HDL synthesis as a result of impaired ABCA1 function may be a factor. The positive relationship between *LXRα*, *ABCG1* and *ABCA1* expression testifies to the important role played by *LXRα* in the regulation of both *ABCA1* and *ABCG1* in humans. Although unsaturated fatty acids negatively regulate *ABCA1* expression *in vitr*o,[12] no relationship was observed with plasma NEFA levels in our study. PPARγ mRNA levels did not significantly relate to *ABCA1* expression in blood leukocytes nor to blood glucose levels. The latter could be due to altered transcription factor activity or modification as PPARγ phosphorylation has been shown to influence the glucose lowering effect of the PPARγ agonist, rosiglitazone.[37] ABCG1 has a major role in cholesterol efflux in humans. However, a human disease caused by ABCG1 dysfunction has not been identified and ABCG1 deficiency in animal studies has not consistently shown evidence of increased atherosclerosis. This questions whether ABCG1 plays an equally critical role in preventing atherosclerosis. Unlike ABCA1, ABCG1 does not have a major role in maintaining plasma HDL-C levels.[38]

Genetic variation in *ABCA1* can lead to functional decline. This is maximal in Tangier disease and observed to a lesser extent in people with other genetic variants.[39] There have been reports that some variants may occur more commonly in T2DM compared to the background population.[40], [41] It is currently unknown whether *ABCA1* genetic variation leads to altered gene expression.

It has been proposed that reduced ABCA1 action in humans leads to impaired β-cell function and reduced insulin secretion has been reported in some Tangier patients.[42], [43] Both ABCA1 and ABCG1 have been implicated in islet cell function in mice with ABCG1 shown to have a central role in insulin secretion.[44], [45] PPARγ agonists may reduce glucose levels in animals through the action of ABCA1 in the β-cell.[44] These data suggest that ABCA1 directly influences glycaemia via its action on β-cell insulin secretion, but other data suggest that it is glucose which modifies ABCA1. Recent animal studies have shown decreased ABCA1 protein and subsequent cellular cholesterol accumulation in macrophages using different mouse models of type I diabetes.[13] This has been observed in other animal models.[46] Hyperglycaemia specifically down-regulates *ABCA1* and *ABCG1* mRNA and protein content in human macrophages in vitro.[21] Similarly, reduction in gene expression has been reported in mouse peritoneal macrophages exposed to high glucose concentrations.[47] In vascular smooth muscle, hyperglycaemia leads to reductions in *ABCA1* mRNA and protein through changes in *ABCA1* promoter activity, possibly via the p38-mitogen-activated protein kinase (MAPK) pathway.[48] These studies, along with our findings in humans, are compatible with a common glycaemia-mediated suppression of ABCA1. There may, nevertheless be reciprocal interactions between ABCA1 and glycaemia as decompensation in glucose metabolism progresses and this is an area requiring future study.

Our results are the first demonstration of a relationship between glycaemia, *ABCA1* expression, ABCA1 protein content and cholesterol removal from cells in humans. Collectively, they suggest that T2DM is associated with impaired cellular cholesterol removal via effects on ABCA1 gene expression and function, impairing the formation of HDL. In humans information pertaining to RCT is limited. Its importance is highlighted by recent observation that the cholesterol efflux capacity of plasma in man has predicted atherosclerosis and coronary disease. [49] Plasma HDL-C levels accounted for only 40% of the variability in efflux and other processes in cellular cholesterol removal are likely to be important.[49] Insights have historically been discovered from the study of rare genetic disorders of ABCA1, where the degree of transporter dysfunction related to the severity of premature atherosclerosis.[34] There has been substantial therapeutic interest generated by work demonstrating improvements in atherosclerosis burden in cardiac patients after infusions of human recombinant Apo-AI (Milano).[50] Moreover, in men with type 2 diabetes, infusion of HDL resulted in short term improvements in cholesterol removal capacity.[51] Whether augmenting cellular cholesterol removal will effectively prevent future cardiovascular events in susceptible patients remains to be proven.

## Supporting Information

Figure S1 **The relationship of leukocyte** ***ABCG1*** **expression to** ***ABCA1*** **expression and to glycaemia.** A. Gene expression dCt = δ cycle number (arbitrary units), rho = 0.40, p = 0.005. B. Normalised gene expression versus HbA1c, rho = −0.29, p = 0.05.
(TIF)

Figure S2 **The relationship of leukocyte Liver X receptor-α (LXRα) gene expression to ABCA1 gene expression and glycaemia (Hba1c).** A. dCt = δ cycle number (arbitrary units), r = 0.40, p = 0.002. B. Normalised gene expression versus HbA1c, rho = 0.02, p = 0.87.
(TIF)

Figure S3 **The relationship of leukocyte peroxisome proliferator-activated receptor-γ (PPARγ) gene expression to ABCA1 gene expression and glycaemia.** A. Normalised gene expression, n = 23,rho = −0.07, p = 0.76. B. Normalised gene expression versus Hba1c, n = 23, rho = 0.31, p = 0.15.
(TIF)
